# Exploring the transcriptomic profile of human monkeypox virus via CAGE and native RNA sequencing approaches

**DOI:** 10.1128/msphere.00356-24

**Published:** 2024-08-27

**Authors:** Gergely Ármin Nagy, Dóra Tombácz, István Prazsák, Zsolt Csabai, Ákos Dörmő, Gábor Gulyás, Gábor Kemenesi, Gábor E. Tóth, Jiří Holoubek, Daniel Růžek, Balázs Kakuk, Zsolt Boldogkői

**Affiliations:** 1 Department of Medical Biology, Albert Szent-Györgyi Medical School, University of Szeged, Szeged, Hungary; 2 National Laboratory of Virology, Szentágothai Research Centre, University of Pécs, Pécs, Hungary; 3 Institute of Biology, Faculty of Sciences, University of Pécs, Pécs, Hungary; 4 Bernhard Nocht Institute for Tropical Medicine, WHO Collaborating Centre for Arbovirus and Hemorrhagic Fever Reference and Research, Hamburg, Germany; 5 Veterinary Research Institute, Brno, Czechia; 6 Institute of Parasitology, Biology Centre of the Czech Academy of Sciences, Ceske Budejovice, Czechia; 7 Department of Experimental Biology, Faculty of Science, Masaryk University, Brno, Czechia; University of Michigan, Ann Arbor, Michigan, USA

**Keywords:** monkeypox virus, CAGE-Seq, nanopore sequencing, long-read sequencing, transcriptome, poxvirus

## Abstract

In this study, we employed short- and long-read sequencing technologies to delineate the transcriptional architecture of the human monkeypox virus and to identify key regulatory elements that govern its gene expression. Specifically, we conducted a transcriptomic analysis to annotate the transcription start sites (TSSs) and transcription end sites (TESs) of the virus by utilizing Cap Analysis of gene expression sequencing on the Illumina platform and direct RNA sequencing on the Oxford Nanopore technology device. Our investigations uncovered significant complexity in the use of alternative TSSs and TESs in viral genes. In this research, we also detected the promoter elements and poly(A) signals associated with the viral genes. Additionally, we identified novel genes in both the left and right variable regions of the viral genome.

**IMPORTANCE:**

Generally, gaining insight into how the transcription of a virus is regulated offers insights into the key mechanisms that control its life cycle. The recent outbreak of the human monkeypox virus has underscored the necessity of understanding the basic biology of its causative agent. Our results are pivotal for constructing a comprehensive transcriptomic atlas of the human monkeypox virus, providing valuable resources for future studies.

## INTRODUCTION

Orthopoxvirus, a genus in the Poxviridae family, encompasses several significant human and animal pathogens. Orthopoxviruses include several species, most notably the variola virus, which causes smallpox, the monkeypox virus (MPXV), the cowpox virus, and the vaccinia virus (VACV), which is known for its use in smallpox vaccination (1–3). Over the course of centuries, smallpox claimed millions of lives until its successful eradication, thanks to an extensive worldwide vaccination initiative (4). MPXV is a zoonotic virus, originally endemic to Africa, that can cause human disease known as mpox (5). Typically, the symptoms are typically mild (6). The first human case was identified in the Democratic Republic of Congo (DRC) in 1970 (7). Since then, sporadic outbreaks have been increasingly reported in Eastern, Central, and Western Africa (8). Human MPXV (hMPXV) is classified into three phylogenetically distinct clades: clade I, the most virulent, with up to 10% human mortality, primarily transmitted by rodents in the Congo Basin (9); clade IIa, originally a zoonosis with low mortality in West Africa, which evolved into human-to-human transmission, causing an outbreak in Nigeria in 2017–2018 (9, 10); and clade IIb, responsible for the 2022 global outbreak, spreading through human-to-human transmission (9, 11, 12). The existence of multiple subclades indicates rapid evolution of these human-adapted lineages (9, 11, 12).

Orthopoxviruses have a large linear double-stranded DNA genome, approximately 200 kilobase pairs long (13). Unlike the majority of mammalian DNA viruses, including herpesviruses and adenoviruses, which replicate in the nucleus, poxviruses, along with the African swine fever virus, replicate in the cytoplasm. The replication and transcription processes of poxviruses are carried out within specialized structures known as “viral factories” (14). The regulation of viral gene expression is governed by transcription factors specific to different stages, which bind selectively to the promoters of early (E), intermediate (I), and late (L) genes (15). The full transcription machinery is pre-packaged within the poxvirus virion, which allows for immediate expression of E genes once the virus has entered the cell and while the viral genome is still encapsulated. This is then followed by DNA replication and the subsequent expression of I and L gene classes, collectively termed as post-replicative (PR) genes. E genes are responsible for encoding proteins that synthesize DNA and RNA molecules and those that play a part in the interactions between the virus and the host. Meanwhile, PR genes primarily encode the structural elements of the virus (16).

Unlike herpesviruses, which tend to produce 3′-co-terminal transcripts by the adjacent tandem genes, poxviruses generate a vast diversity of 3′-ends (17), especially during the late stages of infection (18). The lack of splicing in poxvirus transcripts is attributed to their replication in the cytoplasm (19). Poxviruses have the unique ability to produce their own enzymes for capping, decapping, and polyadenylation, and they employ strategies such as mRNA decapping to inhibit host translation (20). Though poxvirus mRNAs generally resemble host mRNAs in structure, one distinctive trait is the presence of 5′-poly(A) leaders in PR mRNAs (21). Recent studies have revealed that poly(A) leaders provide the capability to utilize either Cap-dependent or Cap-independent translation initiation (22).

Several studies have explored the transcriptional impact of hMPXV infection across various cell types, predominantly utilizing micro-array-based techniques (23–26). These pioneering works have laid the foundation for the understanding of the viral transcription landscape. A notable limitation of the micro-array-based techniques is their inability to resolve important aspects of the transcriptome, particularly to detect the transcript isoforms (27). Determining the exact genomic location of the transcription start sites (TSSs) and transcription end sites (TESs) of the mRNAs is crucial in annotating viral genomes. Methods, such as S1 nuclease treatment with labeled probe-hybridization (28, 29), have been developed as early attempts to determine both 5′-ends (30–34) and 3′-ends (35–37) of poxviral mRNAs. Rapid amplification of cDNA ends (RACE) (38) is a widely used PCR-based method to identify ends of cDNA transcripts. This technique was used to determine transcript boundaries in poxviruses (39). Poxviruses are unique among the viruses, because they have their own capping (40) and decapping enzymes (41, 42). Detection of Cap is utilized in transcriptome research for identifying transcription initiation (43, 44). Although microarray and PCR-based techniques offer high precision, they are limited to analyzing only those transcripts for which probes or gene-specific primers exist. In contrast, total RNA-sequencing methods allow for the examination of the entire transcriptome. With the advent of next-generation sequencers, the bulk analysis of whole transcriptome features, including TSSs and TESs, became possible.

Various sequencing methods are available to infer the whole transcriptome. Short-read sequencing (SRS) provides a high-throughput, base precision map of transcriptional activity. However, reverse transcription-dependent techniques are unable to circumvent the drawbacks occurring during cDNA synthesis, such as template switching (45, 46), false priming (47), or spurious antisense transcription (48).

Long-read sequencing (LRS) methods, such as single-molecule real-time and nanopore sequencing (49, 50), are able to read the entire mRNAs, making them indispensable in transcriptome research (51, 52). Oxford Nanopore Technologies (ONT) allows for the direct sequencing of native RNA molecules (dRNA-Seq). This approach eliminates the generation of false products that may arise during the library preparation process, specifically during the reverse transcription, second-strand synthesis, and PCR steps. The limitation of this technique is its reduced precision in annotating the 5′-ends of mRNAs (53). This issue can be mitigated by integrating dRNA-Seq with 5′-end-sensitive PCR-free direct cDNA (dcDNA) sequencing (dcDNA-Seq) or selective detection of capped RNA ends by Cap Analysis of Gene Expression sequencing (CAGE-Seq) methods (54–56). The LRS cDNA-Seq approach has been applied for the analysis of dynamic VACV transcriptome (18, 27, 57, 58). Host cell transcriptome was recently inferred upon hMPXV infection (59). However, the transcriptome of hMPXV itself has not been analyzed.

Our objective in this study was to identify TSSs and TESs of viral mRNAs, which helps annotate the complete viral transcriptome. Furthermore, we identified the promoter and poly(A) signal consensus elements of the hMPXV genes.

Genome sequencing studies are essential for tracking genetic mutations during a viral outbreak, while transcriptomic analyses are necessary to discover novel genes. These analyses offer insights into gene expression patterns and isoform variations in poxviruses. Notably, non-conserved regions such as the inverted terminal repetition (ITR) segments undergo rapid microevolution (8, 60). These regions are crucial for determining host spectrum and evading host immune responses, making their study critically important. Despite a recent decline in clade IIb mpox cases, the risk of a future outbreak should not be underestimated. In 2023, the number of suspected hMPXV infections increased in the DRC, with genetic analysis indicating that clade I viruses, transmitted through sexual contact—a characteristic previously only described for clade IIb—are responsible (61).

## RESULTS

We employed two distinct sequencing approaches to identify the terminal regions of the hMPXV transcripts. TSSs were detected using CAGE-Seq on the Illumina MiSeq platform, whereas TESs were identified through dRNA-Seq on the ONT MinION device.

### Transcription start sites

**Fig 1:**
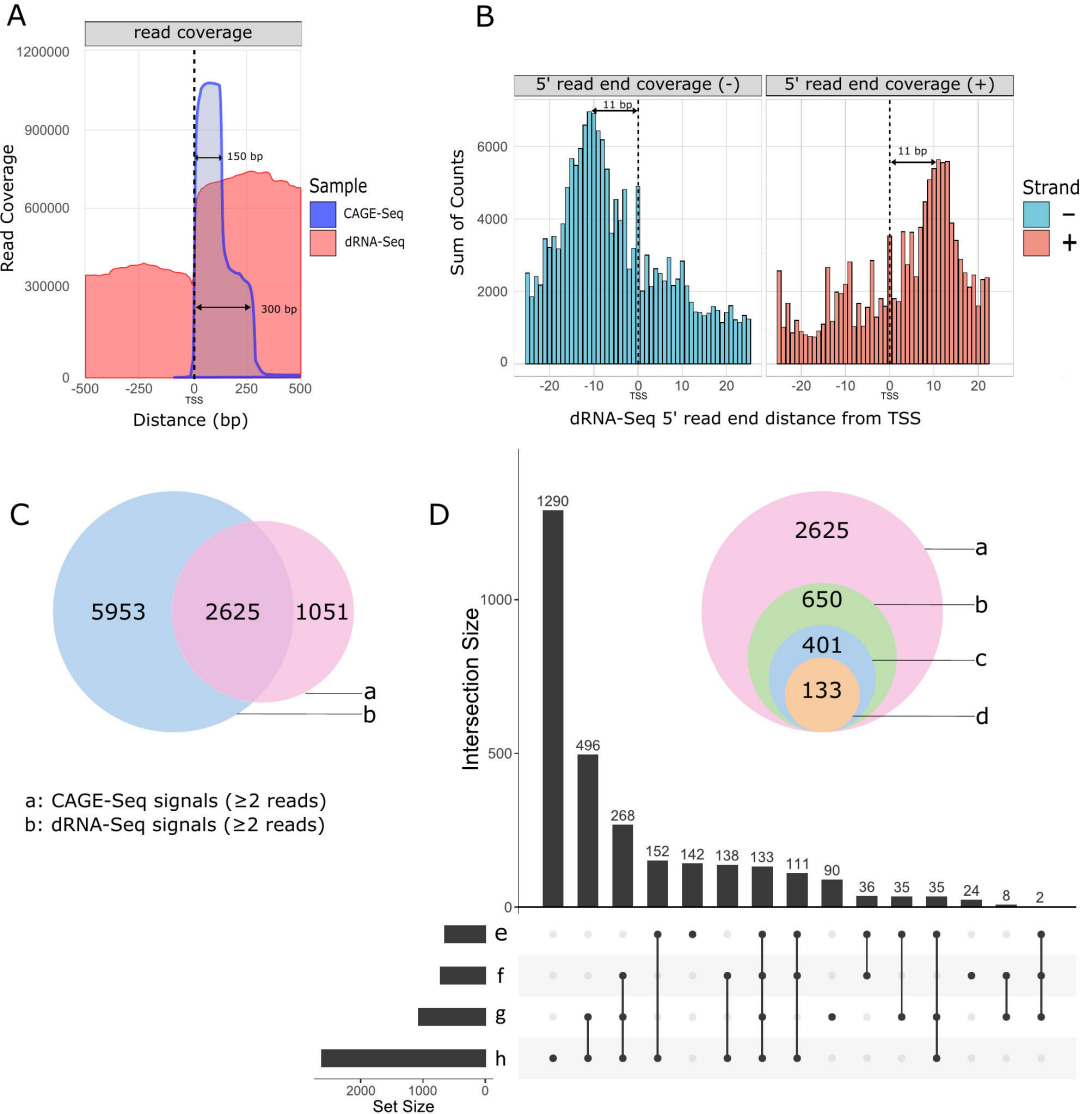
Distribution and characterization of 5′-ends of hMPXV mRNAs. (**A**) The figure shows the raw read coverage of all superimposed CAGE-Seq and dRNA-Seq reads around all annotated TSSs (dashed black line represents the position of TSSs). The x-axis represents the distance from the TSS, while the y-axis indicates the coverage. The CAGE-Seq is a composite of 150-bp and 300-bp libraries. The figure demonstrates that the coverage of dRNA-Seq and CAGE-Seq reads generally agrees, providing a clear signal for detecting transcriptional start positions. (**B**) The histogram illustrates the distribution of 5′-ends of dRNA-Seq reads around all CAGE-Seq TSSs in a ±25-nt window. The x-axis represents the distance from the TSS, while the y-axis indicates the sum of read counts. The dRNA-Seq 5′-ends most frequently accumulate 11 nt downstream from the TSSs, which is seen as two dominant peaks on the histogram. (**C**) Venn diagram shows the initial number of putative TSSs in CAGE-Seq and dRNA-Seq and their intersection before applying the filtering criteria. (**D**) The upset plot illustrates the intersections between different subsets of our data during the filtering process. The subsets are defined based on specific criteria applied to the CAGE-Seq and dRNA signals. The onion diagram depicts the number of CAGE-Seq signals according to the filtering method implemented in this study. (a) All detected CAGE-Seq peak signals, except singletons, corroborated by dRNA 5′-ends located within a 25-nucleotide window downstream from the TSS. (b) Number of CAGE-Seq peaks with at least 10 read counts corroborated by dRNA 5′-ends located within a 25 nucleotide window downstream from the TSS. (c) CAGE-Seq signals with at least 10 read counts, corroborated by a promoter motif detected within a 40-nucleotide interval and co-terminating with dRNA-Seq reads within a 25-nucleotide window downstream from the TSS. (d) Number of dominant TSS signals within the clusters of CAGE-Seq signals that match the filtered TSS data. (e) Clusters of CAGE-Seq signals that are the dominant peaks within their cluster. This subset focuses on the most prominent TSS within each cluster, indicating the primary sites of transcription initiation. (f) Set of CAGE-Seq signals that have a count value of at least 10. This subset is used to highlight robust CAGE-Seq signals that are likely to represent significant transcription start sites. (g) CAGE-Seq signals that have a promoter element within 40 nucleotides upstream. This subset helps identify TSSs that are located near promoter elements, providing insights into promoter activity. (h) CAGE-Seq signals that are validated by a dRNA 5′-end within 25 nucleotides. This subset indicates the proximity between CAGE-Seq and dRNA-Seq data, suggesting high-confidence TSSs.

**Fig 2:**
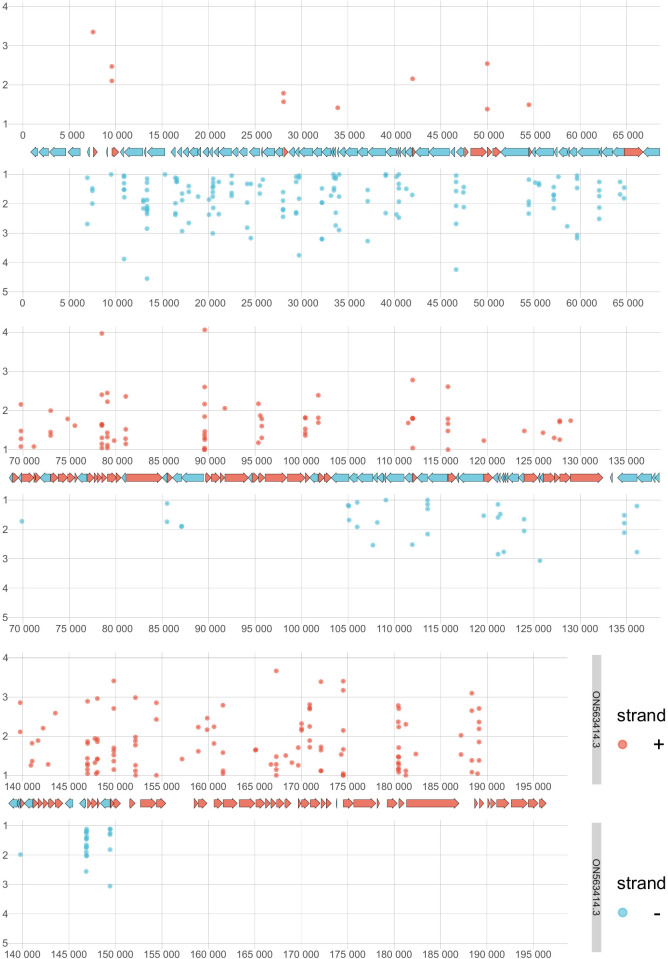
Distribution of filtered TSSs along the viral genome. The figure illustrates the annotated genome of hMPXV (ON563414.3), depicting the positions of TSSs determined by CAGE-Seq. We applied specific filtering criteria to identify these TSSs: a minimum of 10 CAGE-Seq signals at a position, a predicted promoter motif within a 40-nucleotide window upstream of the TSS, and at least one dRNA-seq 5′-end with a minimum read count of 2 within a 25-nucleotide window downstream from the TSS. This resulted in a total of 401 TSSs. TSSs on the positive strand are illustrated in red and those on the negative strand in blue. The x-axis denotes the values of CAGE-Seq peaks at each genomic position on a logarithmic scale, and the y-axis denotes the genomic positions.

CAGE-Seq analysis identified a total of 3,676 TSS positions excluding the singletons (Table S1), although dRNA-Seq efficiently validates 5′-ends but encounters challenges due to incomplete sequencing of these termini (53). However, unlike cDNA-Seq techniques, dRNA-Seq is free from common artifacts. Therefore, we opted to utilize this method to validate the results obtained from CAGE-Seq (Fig. 1A; Fig. S1). A total of 2,625 TSSs were confirmed by dRNA-Seq within a 25-nucleotide window, likely representing an underestimate of the overall TSSs. We analyzed the distribution of dRNA-Seq read ends in the proximity of CAGE-Seq signals and found that the 5′-ends detected by dRNA-Seq were most frequently positioned on an average of 11 nucleotides downstream from the TSSs identified by CAGE-Seq (Fig. 1B). The missing nucleotides at the 5′-end result from the premature release of the RNA molecules by the motor protein. We further filtered the 2,625 positions by eliminating those with fewer than 10 supporting reads, resulting in a total of 650 positions by excluding those supported by fewer than 10 reads (Fig. S2). Subsequently, we analyzed which of these positions were within 40 nt upstream of a predicted promoter. This latter analysis yielded a final count of 401 positions (Fig. 2).

Furthermore, employing another novel TSS clustering algorithm within the TSSr package (peakclu), we identified 646 clusters of CAGE signals, each with a single dominant peak (Table S2). Comparing these dominant peaks of the clusters with the data set of 401 filtered TSS positions, we identified a set of CAGE signals comprising 133 positions that met all the filtering criteria (Fig. 1C and D; Table S2).

**Fig 3:**
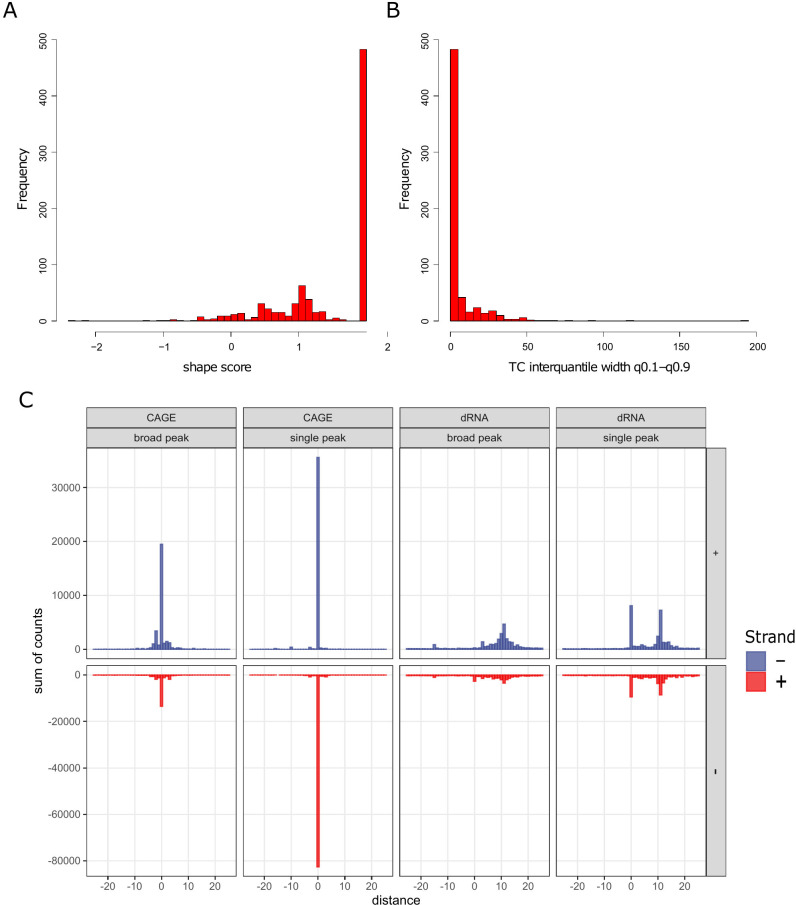
Cluster analysis of CAGE-tags by TSSr. (**A**) Histogram of Shape Index (SI) scores of TSSs. Higher SI values indicate sharper core promoters, with an SI value of 2 corresponding to a single peak per cluster. (**B**) The histogram displays the distribution of inter-quantile widths of TSS clusters in TSSr. The majority of peaks occurred within a 50-nt distance around a given TSS. (**C**) Histogram of 5′-ends around TSSs, according to the two types of TSS clusters within a 50-nt distance in the two libraries (dRNA-Seq and CAGE-Seq). Broad-range clusters feature a wider distribution of TSSs, whereas single-peak clusters exhibit a more concentrated distribution of TSSs. TSSs are grouped together based on their shape values. The dRNA-Seq reveals an 11-nucleotide shift in the accumulation of 5′-ends, accompanied by a distinct single peak indicating that a portion of the reads has been completely sequenced.

This shows that both clustering and unclustering of CAGE signals lead to robust TSS detection, demonstrating their consistency. Using the shape score index, peak analysis of CAGE-Seq data revealed two major types of TSS distributions: broad and narrow range. The analysis indicated that the majority of the clusters consist of single peaks, with the vast majority the clusters not surpassing 10 nt in width (Fig. 3).

**Fig 4:**
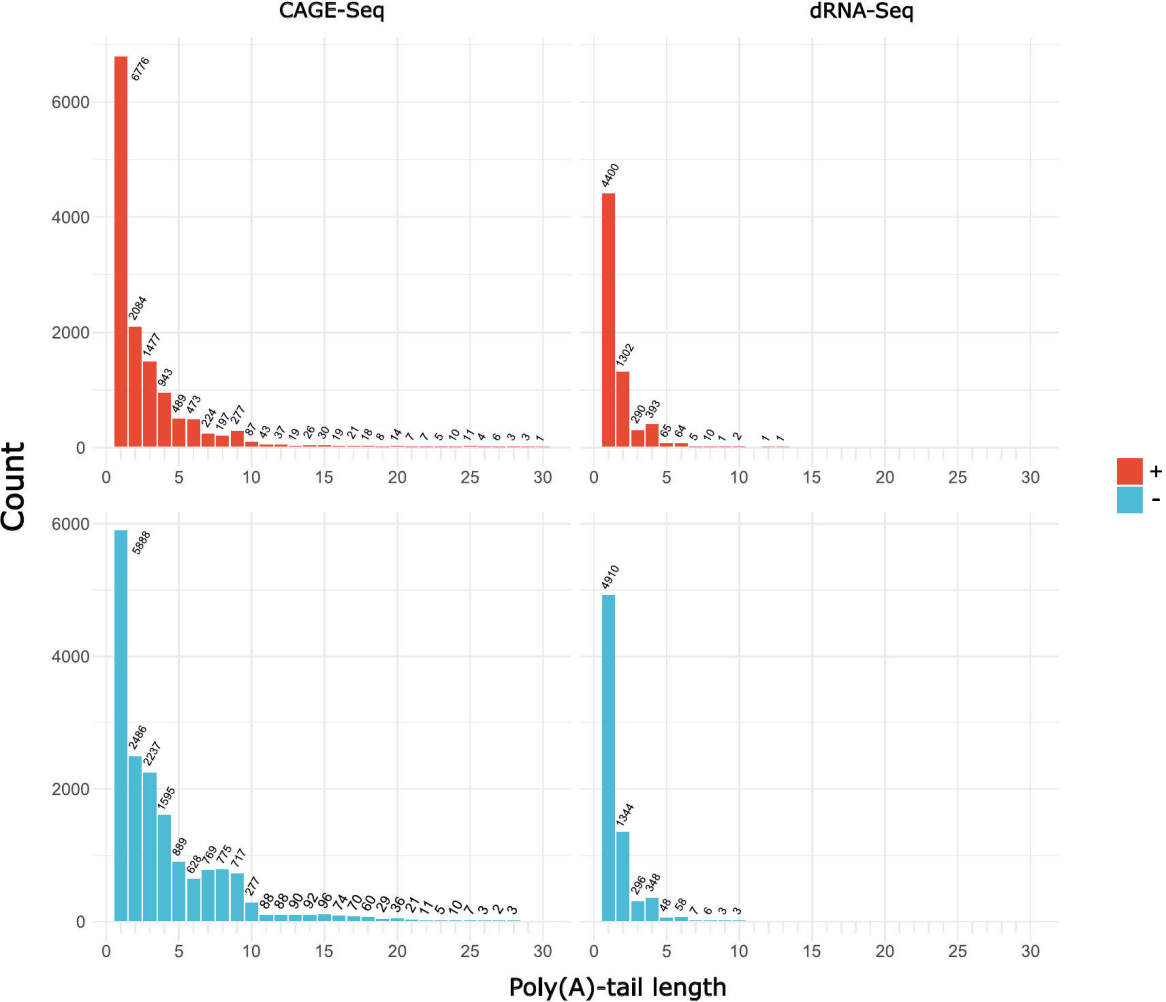
Distribution of the length of the 5′-poly(A) leader sequence in hMPXV. The distribution of the length of the 5′-poly(A) leader sequence in CAGE-Seq and dRNA-Seq samples from both the + and − strands. The x-axis denotes the length of the poly(A) leader (excluding values of 0), while the y-axis represents the number of reads.

The distinguishing characteristic of poxviral mRNAs is the presence of poly(A) leader sequences at the 5′-ends of late mRNAs (62, 63). Despite the absence of 11 nt on average at the 5′-end of dRNA-Seq reads, the presence of a 5′-poly(A) leader enables the sequencing of the entire molecule, as shown in Fig. 3C. We estimated the number of 5′-poly(A) leaders and found that 10% of CAGE-Seq reads and 5% of dRNA-Seq reads contain at least three A bases (Fig. 4).

TSS positions were sorted according to their abundance. The top five TSSs surpass a read depth of 1,000 in CAGE-Seq (Table S3). Among these, three TSSs stand out with exceptionally high CAGE-Seq signals, each showing count values exceeding 10,000. The highest CAGE-Seq signal represents 13% of the total and nearly 44% of the count for the top five TSSs. In dRNA-Seq, the gene OPG110, which encodes the protein VLTF-4 involved in post-replicative transcription elongation of L genes, has the most abundant 5′-end position. Out of the most abundant 5′-CAGE-Seq positions, three coincided with the most abundant dRNA-Seq positions belonging to the following genes: OPG065, OPGOPG110, and OPG022. Table S3 provides a summary of the orthologs and functions of genes associated with the most abundant TSS positions.

### Promoter elements

Our understanding of promoter elements in orthopoxviruses primarily stems from research on VACV (64, 65). Poxviruses use distinct promoter motifs in the early and late phases of infection (66). Given the close phylogenetic relationship between VACV and hMPXV (67, 68), the promoter motifs of the former virus were employed to identify corresponding elements in hMPXV (Table 1).

**TABLE 1:** Cis-regulatory sequences used for promoter and PAS annotation^*a*^

Type	Kinetics	Consensus	Reference
Promoter	Early	AAAANTGAAAANNA	56
	Late	TAAATG/NNNTNNNNNNNNNTAAATG	69; Yang et al. (56)
	Group I	NNNNNNNNNNYNWNWWWTWWWNNNNNWTAAATG	Yang et al. (56)
	Group IIB	NATWWNWNNNHTAAAAANNDNNNNHNNDWWNTAAAYN	
	Group IIA	NRNNWNWTNWMWNWWWWTDNNNNH	
	Intermediate	NNNATNNNNNNNNTAAAAANNNNNNNNNNNNNNTAAA	70
	Mixed	NNNA/TNNNNNNNNTNNNNNNNNNTAAATGGNNN	Yang et al. (56)
	Mixed	NTAWAD	Tombácz and Prazsák et al. (57)
PAS	Early	UUUUUNU	Yang, Reynolds et al. (70); Yang et al. (16)

^*a*^ The promoter motifs used to scan viral promoter and PAS sequences are categorized by their kinetics, based on data from literature on experiments related to VACV gene expression. PAS stands for poly(A) signal.

**Fig 5:**
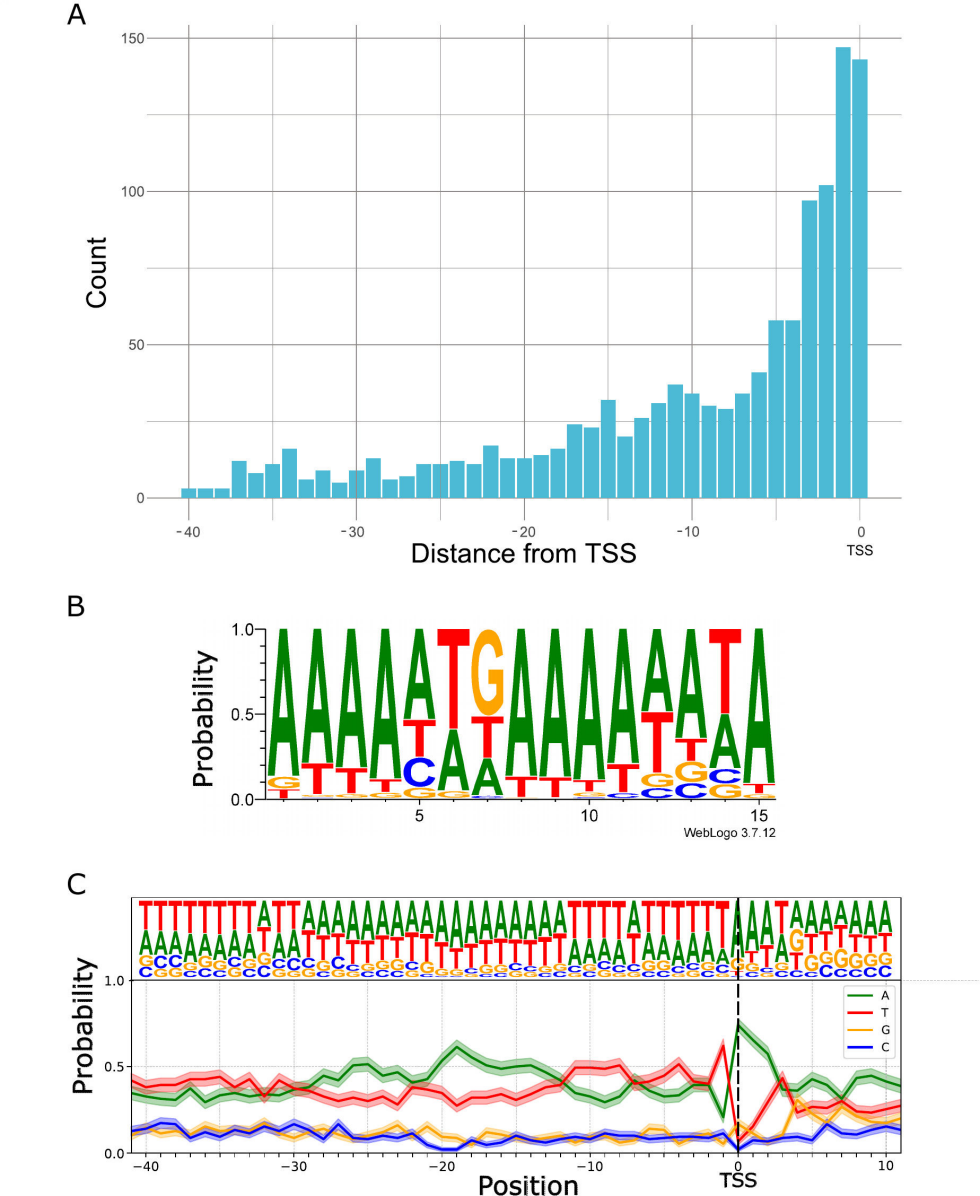
Promoter elements in hMPXV genome. (**A**) Distribution of promoter motifs within a 40-nt interval upstream of TSSs. (**B**) The consensus motifs of early promoters are illustrated by WebLogo. (**C**) Base composition probability near TSSs associated with post-replicative promoters. The TSS within the conserved TAAAT motif is indicated by dashed line.

We identified 1,369 putative promoters within a 100-nt interval upstream of TSSs using the FIMO (Find Individual Motif Occurrences) program. The resulting predicted promoters, along with their *P* and *q*-values, are listed in Table S1c. The best-matching motifs, associated with the names of ORFs, are organized according to their *q*-values and detailed in Table S1d. The average distance between each TSS and its predicted promoter motif was determined to be 26 nucleotides, with the most frequent distance observed being 1 nucleotide (Fig. 5A). This finding is consistent with results from studies conducted on VACV (56).

### Transcription end sites

**Fig 6:**
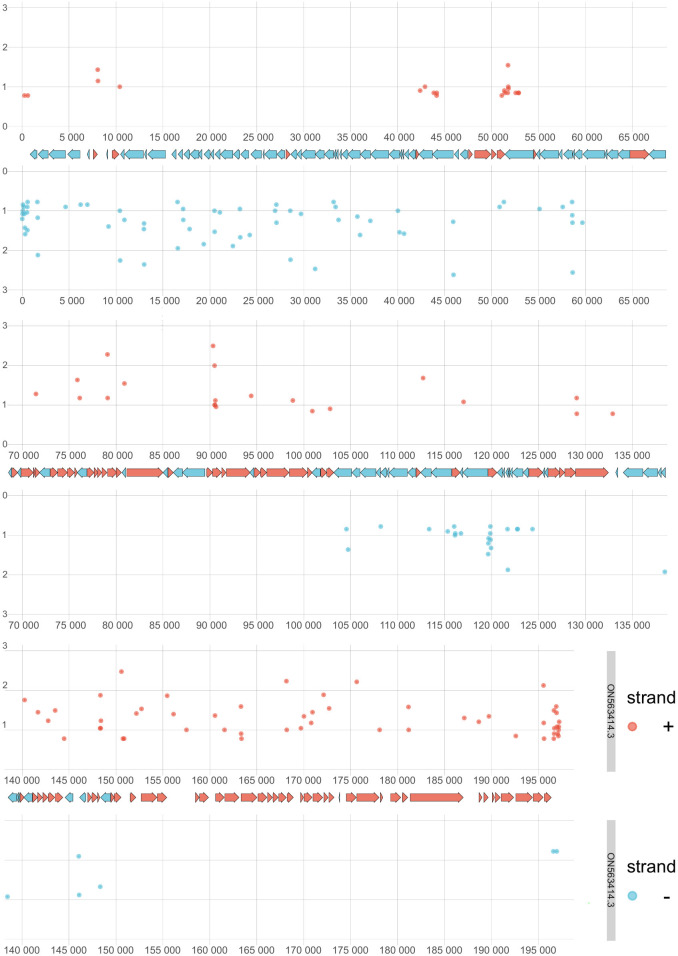
Distribution of filtered TESs. The figure displays the global distribution of TES positions with a minimum count of six in the dRNA-Seq data. The x-axis represents the count on a log10 scale, while the y-axis indicates the genomic position.

Direct RNA sequencing, based on poly(A) selection, was employed to identify the 3′-ends of hMPXV RNAs, using the LoRTIA (71) tool for TES annotation. A total of 3,241 positions were identified (excluding singlets), with 496 of these positions validated by a minimum of six reads (Fig. S3). Among these, 135 positions were further validated by ePAS signals within a 50-nucleotide distance (Fig. 6; Table S4).

### Poly(A) signals

**Fig 7:**
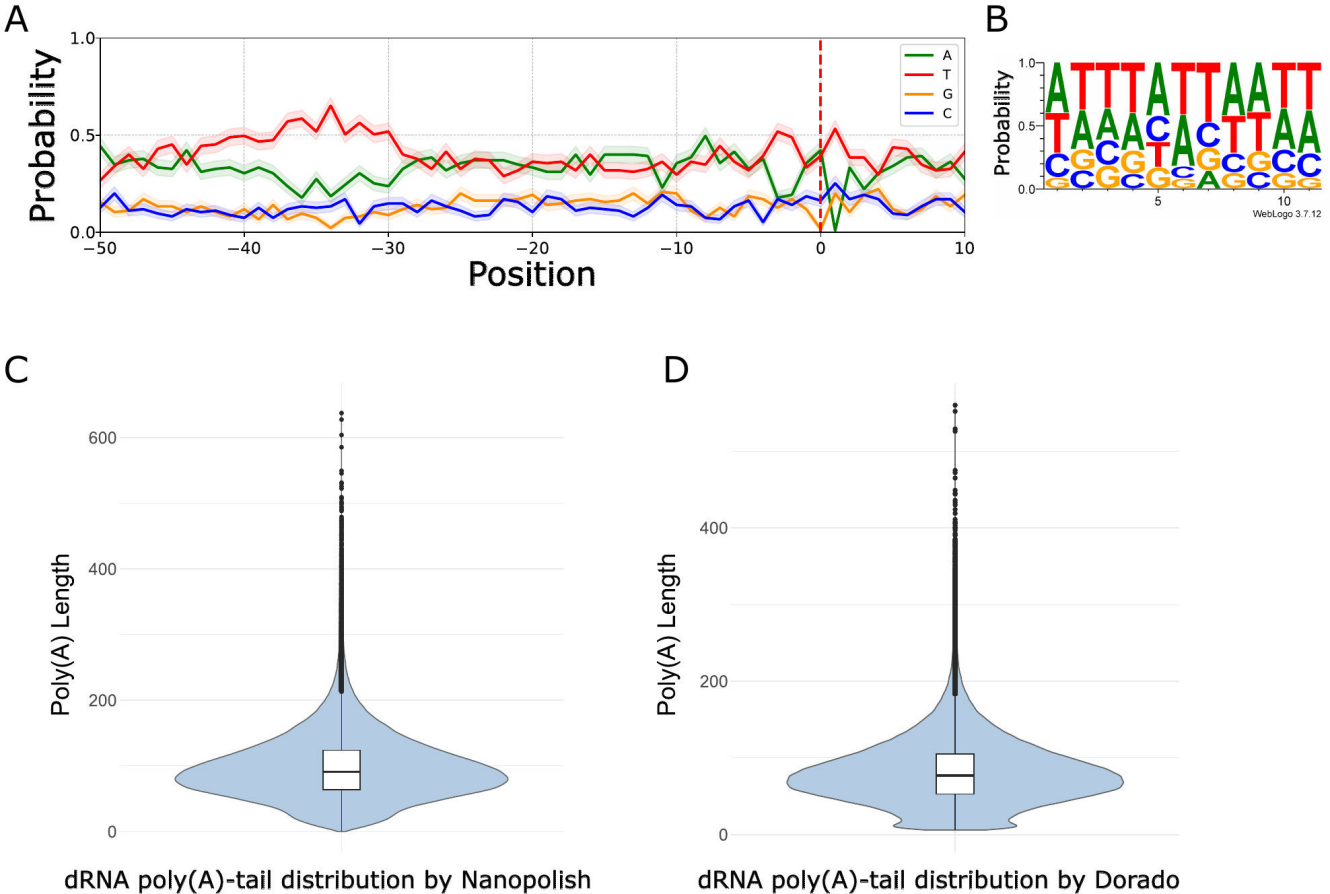
Characterization of TESs and poly(A)-tails of hMPXV mRNAs. (**A**) The PASs of the early ORFs are located within 50 nt upstream of the TESs, which are represented by a dashed red line. (**B**) The TES is characterized by a dominant A/T nucleotide composition. (**C**) The poly(A)-tail length distribution of viral dRNA-Seq reads estimated by Nanopolish. (**D**) The poly(A)-tail length distribution of viral reads estimated by Dorado.

Orthopoxviruses utilize their unique enzymatic machinery to recognize polyadenylation signals (PASs) and to synthesize the poly(A)-tail of viral mRNAs. VACV early mRNAs are characterized by a UUUUUNU early PAS (ePAS), leading to a premature and homogenous end of early mRNAs (56, 72). Using a motif scanning algorithm (FIMO), we identified 734 ePASs, as detailed in Table S4. Of these, 313 ePASs were found 50 nt upstream of TESs, validating 135 of the previously mentioned 496 TESs, as reported in Table S4. The average distance of ePAS from TESs is 24 nt, which is in concordance with VACV data (56, 70). One benefit of dRNA-Seq is its ability to directly analyze the native poly(A)-tails of RNAs. In the analysis of 232,258 hMPXV mRNAs, the mean poly(A)-tail length was found to be 97.91 nt (with an SD of 51.07 nt) according to Nanopolish and 82.21 nt (with an SD of 43.48 nt) as measured by Dorado. The most frequent poly(A)-tail lengths were 86 nt and 71 nt (Fig. 7; Table S5).

### UTRs of hMPXV genes

The hMPXV genome displays the densely packed and sequentially arranged gene structure common to orthopoxviruses. This layout creates many short intergenic regions, with an average distance of 129 nucleotides between genes, which often causes the untranslated regions (UTRs) of neighboring genes to overlap. Following the annotation of TSSs and TESs, we identified the canonical UTR for each ORF in hMPXV. To determine the 5′-UTRs, we initially aligned the filtered TSS positions with the coordinates of a given ORF and selected the most abundant closest TSS as canonical.

We found that 118 out of 190 ORFs had an associated TSS, while the remainder either failed to meet our strict criteria or shared a common TSS with other ORFs. The length of the canonical 5′-UTRs ranged from 0 to 590 nt, with an average of 72 nt (see Table S6a). This excludes cases where the TSS was located within the host ORF. The 5′-UTRs can also be distinguished by their TSS distribution. We discovered that 63 ORFs have a single, highly abundant TSS, while 55 ORFs could be associated with a non-single peak type of TSSs. Additionally, 20 TSSs were found in the upstream neighboring ORF, and 7 TSSs were detected within the host ORF (Table S6b). The dRNA-Seq facilitates the identification of complete transcript boundaries. We observed that many ORFs have alternative transcription initiation sites, often including one or more upstream ORFs. We identified these low-abundance 5′-UTRs, where one or more ORFs are covered by the UTR of a downstream ORF, as detailed in Table S6a. It is known that VACV produces heterogeneous 3′-ends (16, 27); therefore, determining the length of 3′-UTRs is challenging. We examined the 3′-UTRs of hMPXV based on the closest TES to a given ORF and found that out of 190 ORFs, 113 are assigned to TESs. The mean length of 3′-UTRs was found to be 176 nt. According to our data, almost half of the canonical 3′-UTRs are terminated in the downstream ORFs (Table S7).

### Putative novel genes

An in-depth analysis of TSS positions showed CAGE-Seq signals within intergenic spaces located at the variable ends of the genome. These signals, identified in both the right and left terminal regions, were validated by the ends of dRNA-Seq reads (Table 2).

**TABLE 2:** List of novel TSS and TES positions in intergenic region of hMPXV^*a*^^,^*^b^*

TSS (CAGE)	TES (dRNA)	Strand	Localization	Promoter start	Adjacent downstream gene	Adjacent upstream gene
6,936	6,230	−	LTR	6,930; 6,949	OPG018	OPG015
9,501	9,203	−	LTR	9,504	OPG020	OPG021
152,144	152,157	+	RTR	152,117	OPG178	OPG180
157,160	157,506	+	RTR	157,126	OPG181	OPG184
168,981	169,692	+	RTR	168,951	OPG195	OPG197
187,189	187,794	+	RTR	187,160	OPG210	OPG214

^*a*^ Novel TSSs and TESs have been identified in both the left and right variable regions of the hMPXV genome. Their positions were determined based on sequence alignment against the first public hMPXV reference sequence (ON563414.3) from the 2022 outbreak (73). The locations of the TSSs are indicated as follows: left terminal region (LTR) and right terminal region (RTR). The possible lengths of ORFs are calculated by taking the coordinates from the first ATG to the following STOP codon, along with the dRNA-seq reads.

^*b*^ LTR= left terminal region, RTR= right terminal region.

**Fig 8:**
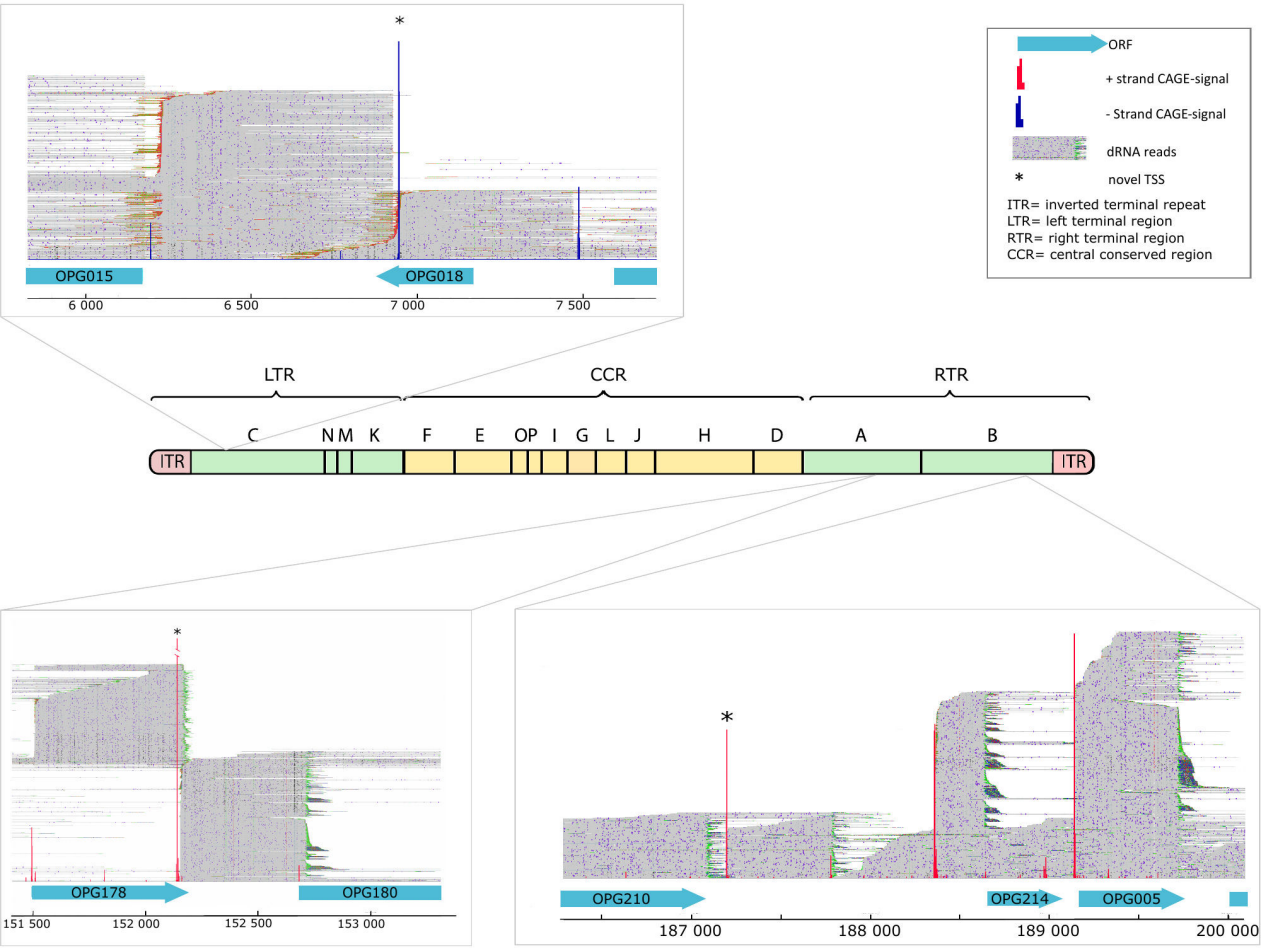
Novel hMPXV genes. The figure shows the localization of the three most abundant novel genes in the hMPXV genome. These putative novel genes are located within intergenic positions. ORFs are indicated with blue boxes in both the right and left terminal regions of the genome. Novel TSSs are indicated by asterisks. The dRNA reads visualized in IGV reveal a novel gene located between OPG018 (MA001-005/D2L) and OPG015 (MA001-004) at the left terminal region. A novel TSS is located between the ORFs OPG178 (MA001-158/Thymidylate kinase) and OPG180 (MA001-159/DNA-ligase) and downstream of the OPG210 (MA001-182/B21R) gene in the right terminal region of hMPXV. The letters above the genome indicate the HindIII fragments of hMPXV (source: ViralZone).

The new genes were further corroborated by the prediction of promoter elements and by dRNA-Seq identifying their TESs (Table S8). Three of the most abundant novel TSSs are demonstrated on Fig. 8.

Despite their short predicted ORFs, a pBLAST search revealed homology with poxviral sequences for three entities: a hypothetical ankyrin-repeat containing protein (located between OPG210 and OPG214), a Kelch-like motif containing a possible protein-coding sequence (located between OPG181 and OPG184), and another unknown protein-coding gene situated in the intergenic area of OPG015 and OPG018 (Fig. 8; Table S8).

Nucleotide BLAST was employed to identify orthologs of the novel genes among orthopoxviruses. Five out of six novel genes matched known orthologs of other orthopoxviruses. The exception is found in the intergenic region between OPG020 and OPG021. Detailed results from the nBLAST analysis are provided in Table S8.

### Phylogenomic analysis identifies the MPXV isolate in the B.1 lineage of clade IIb

We performed phylogenomic analysis for the determination of the evolutionary relationship of our isolate. Based on the distance matrix and SNP analysis of the alignment of 44 representative members of MPXVs, our virus isolate belongs to the B.1 lineage of clade IIb (Supplementary File 1).

## DISCUSSION

Although the human monkeypox virus has been known for decades and has sporadically caused periodic outbreaks in Eastern, Central, and Western Africa, the 2022 outbreak has heightened awareness of the virus. Recent studies suggest that the virus is undergoing rapid microevolutionary changes. Within just a few years, both clade I and clade II have evolved the ability to spread from human to human. Therefore, it is crucial to investigate and understand these clades more thoroughly at both the genomic and transcriptomic levels. While many studies have examined hMPXV at the genomic level, they have often missed detecting novel transcriptionally active sites that represent potential new genes. In this study, we employed dRNA-Seq on the ONT MinION platform to identify the precise TESs of hMPXV, known for their considerable diversity in poxviruses (57). Detection of poly(A) signals was used for the validation of dRNA-Seq results. The lrRNA-Seq methods, particularly of the ONT approach, have been found to produce a pervasive 5′-truncation of transcripts, potentially leading to incorrect identification of false TSSs (74). Our previous investigations (18, 75, 76) have also uncovered a diverse range of 5′- and 3′-transcript ends in various viruses, many of which, particularly the TSSs, are likely non-functional or could even be of non-biological origin. To address this issue, we employed CAGE sequencing on an Illumina MiSeq platform, a well-established method for detecting the 5′-ends of capped RNA molecules. While CAGE-Seq is highly reliable, we cannot exclude the possibility that a certain fraction of degraded RNA molecules is also detected by this technique, since it has been shown that mammalian cells contain enzymes in the cytoplasm capable of generating Caps onto uncapped RNAs (77). A key issue is the absence of software capable of unequivocally differentiating genuine RNA molecules from technical artifacts. In light of this, our study focused on the annotation of main transcript ends but also provided data on the low-abundance putative TSSs and TESs.

We compared the 5′-ends of mRNAs from CAGE-Seq libraries, to those generated by dRNA-Seq, and detected that a significant portion of dRNA-read ends is accumulated on an average of 11 nt downstream of a TSSs (Fig. 1B). This discrepancy is mainly due to poor-quality ends of dRNA-Seq reads, which fail to align when local alignment methods are used. To overcome this phenomenon, SRS and LRS methods are combined (78, 79), or adapter ligation is carried out (80).

VACV is the best-studied representative of orthopoxviruses. Since VACV and hMPXV are phylogenetically closely related (81), their promoter motifs are expected to be very similar. Therefore, we scanned the hMPXV genome using a set of VACV promoter modules. The validation of TSSs and TESs was carried out by identifying nearby consensus sequences and poly(A) signals, respectively. We also compiled a list of high-abundance putative transcript ends where cis-regulatory sequences could not be identified nearby. Integration of short- and long-read sequencing data provided a high-resolution map of the viral transcript ends. Extremely high levels of transcriptional activity were detected in both the core and terminal regions of the viral genome. Additionally, we observed mRNA readthrough at the peak of the circularized genome. The positions of the most abundant TSSs, along with their corresponding host ORF, and their VACV orthologs are listed in the Table S3. The most abundant TSS belongs to the gene OPG023, which codes for a short, non-essential protein termed D8L containing an ankyrin-like peptide domain. This domain plays a role in host immune evasion by blocking IL-1 receptors (82) and modulating the NF-κB pathway (83). The second most abundant TSS belongs to the gene OPG065, which might have evolved via episodic positive selection in response to immune selection (68) and host antiviral response (84). In the dRNA-Seq analysis, the most abundant TSS is associated with the hMPXV OPG110 gene, which plays a critical role in replication and for virion morphogenesis (85, 86).

Our findings on the TSS pattern align with previous studies, confirming the existence of two major TSS types: single-peak and broad-range CAGE-Seq signal distributions. Similar patterns have been observed in orthopoxviruses (56), Herpesviruses (79), and other organisms (87, 88). More precise mapping of the TSSs and additional mutagenesis studies are needed to further explore the transcriptomic structure of poxviruses.

Termination of poxvirus transcription requires the interaction between a U(5)NU consensus sequence and the assembly of a ternary complex, which includes the viral termination factor and the RAP94 protein, causing strict 3′-termination of transcripts (89, 90). Unlike early mRNAs, PR RNAs exhibit high heterogeneity in length because the ePAS is unrecognized by the poxvirus transcription termination complex (16, 56). The transcription of orthopoxvirus genes often terminates within the downstream ORFs (16, 56).

Using oligodT selection-based library screening, canonical TES positions were assigned to the annotated ORFs. However, our dRNA-Seq analysis shows that not all ORFs can be assigned canonical TESs due to the presence of TESs likely used by more than one gene in hMPXV. A similar pattern of TES distribution was revealed in VACV using LRS (27, 57), suggesting the formation of co-terminal transcription units. Our LRS method also enabled the annotation of 73 ePAS, confirming the existence of early canonical TESs (Table S4). We detected a 3′-UTR architecture similar to VACV in the hMPXV transcriptome.

We found that the average length of 5′-UTRs in hMPXV is short, consistent with findings reported by others for other orthopoxviruses (56, 57). In some rare cases (Table S6b), anomalous TSSs were located downstream to the annotated start codon, suggesting alternative ATG usage by the virus (27, 84).

It is important to note that UTRs can be influenced by insertion/deletion events. For example, downstream of the OPG201 (MA001-175) gene, only a diffuse PAS is detectable, and the OPG202 (MA001-176) gene lacks a TES. This region contains an [ATAT] repeat, which can disrupt mRNA termination signals (8). However, a detailed analysis of low-complexity regions in our transcriptome revealed that some TESs could not be precisely determined. This could be due to the presence of repeats or the so-called “chaotic” transcriptomic regions, independent of the kinetic class of transcripts, as described in the transcriptomic analysis of VACV-WR (27). The UTR length in poxviruses is influenced by the kinetic expression of a given transcript. It is known that early mRNAs have homogeneous 3′-ends, while post-replicative mRNAs exhibit heterogeneous UTR lengths (16, 56). The presence of 5′-poly(A) leader is a characteristic feature of the poxviral of mRNAs (16). Furthermore, VACV is a cytoplasmic virus, possessing two enzymes (D9, D10) functioning as decapping enzymes in mRNA degradation and translation regulation. In our study, we also detected the poly(A) leaders in both the dRNA and CAGE samples. Although literature suggests an average length of 35 nt for these sequences (91), we observed shorter lengths in hMPXV. However, it is important to consider that these shorter lengths may be underestimations due to the possible incomplete sequencing of the 5′-end.

Direct RNA sequencing confirmed the presence of polyadenylated novel mRNAs in intergenic regions of both the left and right ITRs. These regions of poxviruses are thought to be responsible for host-virus interactions (8); therefore, a similar function is expected for the novel genes. Farlow and colleagues (92) reported mutations in a cidofovir-resistant MPXV strain in the same genomic region. They speculated about the presence of a hypothetical yet-unknown ankyrin-like protein-coding gene which we can confirm here. On the other hand, this virus is classified within the clade II B.1 lineage of hMPXV. Phylogenetic studies show a relatively high mutation rate within this lineage (93, 94). This accelerated evolution is suggested to be driven by the action of the cellular APOBEC3 nucleic acid-editing enzyme in the terminal genomic region (95–97). Genotyping hMPXV via gene or genome sequencing and identifying point mutations are frequently employed to track the pandemic’s progression (12, 98). Several studies have aimed to elucidate the pathogenicity and virulence of hMPXV by examining variations in the terminal region, which encodes proteins involved in immune modulation (99–101). Nonetheless, transcriptomic studies provide the benefit of describing the functional units of the viral genome, rather than merely analyzing gene variants.

The presence of tandem repeats in the ITR regions of poxvirus genomes is well documented (102–104). Desingu et al. identified a region of tandem repeats (AACTAACTTATGACTT) in the 5′-ITR and 3′-ITR regions of the hMPXV (clade IIb B.1) virus, which is absent in other poxviruses (105). Although the function of these unique repeat sequences remains unknown, gene loss and gain have been observed at the ends of the 5′-ITR and 3′-ITR regions among clade I, clade IIa, and clade IIb mpox viruses (106), indicating a continuous mutational hot spot for the virus (105). These changes are accompanied by unique tandem repeats (8).

These regions are located between the ORFs MPXVgp003 and MPXVgp004, positioning them as intergenic and potentially good targets for foreign proteins in vaccine development. However, our long-read dRNA-sequencing data reveal strong transcriptional activity in these tandem repeat regions, suggesting they are not truly intergenic.

In conclusion, we employed advanced sequencing techniques to comprehensively map the transcript ends and cis-regulatory elements of hMPXV. By combining long- and short-read sequencing methods, we accurately identified TSSs, TESs, and their promoter elements. Additionally, we discovered six potential new genes, significantly updating the genic and intergenic annotation of hMPXV. Our findings underscore the importance of ongoing transcriptomic exploration in infectious disease research, emphasizing the need for further studies to elucidate the dynamic transcription profile of the virus and its complex interactions with the host.

## MATERIALS AND METHODS

### Virus propagation and RNA isolation

The methods for cell culture, virus propagation, and RNA isolation are detailed in the Supplemental Text. Briefly, the hMPXV isolate was propagated in CV-1 cell lines at a multiplicity of infection (MOI) of 5, with three replicates, in 75-cm² flasks. The infected cells were then incubated at 37°C for 2, 6, 12, and 24 hours. RNA was isolated using the Nucleospin RNA Mini Kit (Macherey Nagel) according to the manufacturer’s protocol at each time point, followed by DNase treatment to remove residual DNA. Thereafter, polyadenylated RNA enrichment was carried out using Lexogen’s Poly(A) RNA Selection Kit V1.5. RNA samples were bound to beads, washed, and hybridized. After incubation and washing, the polyadenylated RNA was eluted in nuclease-free water and stored at −80°C for subsequent analysis.

### Native RNA sequencing

The Oxford Nanopore Technologies SQK-RNA002 Kit was utilized to sequence the RNA molecules. For library preparation, we used 50 ng (in 9 µL) of a pooled sample of poly(A) ^(+)^ RNAs. The initial step involved the ligation of a 1-µL RT Adapter (110 nM; part of the ONT Kit) to the RNA sample using a mix of 3 µL NEBNext Quick Ligation Reaction Buffer (New England BioLabs), 0.5 µL RNA CS (ONT Kit), and 1.5 µL T4 DNA Ligase (2 M U/mL, New England BioLabs). This process was conducted at room temperature (RT) for 10 minutes. Subsequently, the cDNA strand was synthesized using SuperScript III Reverse Transcriptase (Life Technologies), with the reaction taking place at 50°C for 50 minutes, followed by a 10-minute inactivation phase at 70°C. After this, the sequencing adapters from ONT’s DRS Kit were ligated to the cDNA at RT for 10 minutes using the T4 DNA ligase enzyme and NEBNext Quick Ligation Reaction Buffer. The final direct RNA library was sequenced on an R9.4 SpotON Flow Cell. To wash the direct RNA-seq and direct cDNA-seq libraries after each enzymatic reaction, RNAClean XP beads and AMPure XP beads (both sourced from Beckman Coulter) were employed.

### Cap analysis of gene expression

The detailed protocol is described in the Supplemental Methods. Briefly, to investigate TSS patterns in hMPXV, we used CAGE-Seq. Total RNA (5 µg) was prepared into CAGE-Seq libraries, starting with RNA denaturation and first-strand cDNA synthesis using the CAGE Preparation Kit. Post synthesis, the RNA was oxidized, and biotin was attached to the 5′-Cap. Biotinylated RNA underwent Cap-trapping on Streptavidin beads, followed by sequential washing and cDNA release. The capped cDNAs were isolated and treated with RNase to remove residual RNA. Streptavidin beads were prepared and washed, and linkers were attached to the cDNAs. After ligation, samples were treated with Shrimp Alkaline Phosphatase and USER enzyme to prepare for second-strand cDNA synthesis. Following synthesis, the samples underwent multiple purification steps and were sequenced on an Illumina MiSeq instrument. The sample concentration and library quality were assessed using Qubit 4.0 and TapeStation, ensuring accurate transcription start site profiling. The CAGE sequencing was performed on the MiSeq platform with the v2 (using 150 cycles) and v3 (using 300 cycles) reagent kit.

### Bioinformatics

#### CAGE sequencing analysis

The reads derived from CAGE-Seq were mapped using STAR to the reference genome with the following parameters: *STAR --runThreadN 8 --outSAMunmapped Within --alignIntronMax 1000*. The bam files were merged after mapping into one dataset (Fig. S4). The downstream analysis was conducted within an R environment (version: 4.2). Due to technical artifacts and stochastic transcriptional activities, TSSs inferred from CAGE-Seq may not represent *bona fide* TSSs. Therefore, we applied the TSSr program (https://github.com/Linlab-slu/TSSr) for CAGE-Seq signal analysis, which effectively handles this problem (107). As one function of TSSr did not work properly, we removed the soft-clips from the alignments using the script at GitHub (https://github.com/gabor-gulyas/softclipremover). The *getTSSs* function was used with two sets of parameters: one for the core region and one for the repeat regions. In the core region, default parameters were used; however, the threshold for the mapping quality in the terminal repeats needed to be decreased (*mapq* ≥ 3) to include the secondary alignments that have lower values. The distribution of CAGE-signals has been calculated by the SI score of TSSr’s *shapeCluster* function. TSS clusters were identified by the “*peakclu*” algorithm in TSSr. The *clusterTSS* function calculates the inter-quantile width of TSS clusters based on the cumulative distribution of CAGE signals. At least 80% of CAGE signals within a cluster was defined as the 5′-and 3′-boundaries of the TSS clusters (107).

#### Long-read direct RNA sequencing analysis

During sequencing, the reads generated were basecalled using the fast model of the Guppy program (https://community.nanoporetech.com). We performed the mapping using Minimap2 (version: 2.17-r941) with the following parameters: minimap2 -ax splice -Y -C5 -t4 --cs. The reference genome was downloaded from NCBI GenBank (accession: ON563414.3) (73). Furthermore, we used the LoRTIA pipeline, developed in our laboratory, for assessing sequencing adapter quality and poly(A) sequences. It also helps eliminate false TESs that could arise from several sources, as described earlier (47). To ensure the alignments were not results of internal priming events, we applied the talon_label_reads submodule of the TALON software package (108).

The LoRTIA program (https://github.com/zsolt-balazs/LoRTIA) was used with the following parameters: *LoRTIA five_score = 16.0, three_score = 16.0 three_adapter='AAAAAAAAAAAAAAA', five_adapter='GCTGATATTGCTGGG'* to identify 5′- and 3′-adapters on the sequencing reads and to determine the TES positions. To estimate the length of polyA tails of viral native RNAs, two methods were used: (i) Nanopolish (https://github.com/jts/nanopolish) using the polyA command with default parameters and (ii) Dorado (https://github.com/nanoporetech/dorado) using the following parameters: *--estimate-poly-a --min-qscore 6*.

#### Identifying the promoter elements and poly(A) signals of hMPXV

These sequence elements were identified using FIMO (109). For promoter identification, the following command was used: *fimo --oc . --verbosity 1 --bgfile --nrdb-- --thresh 1.0E-4 motifs.meme ON563414.3.fasta*, while for PAS identification, the same command was used with the exception of lowering the threshold to 10^−3^ (*--thresh 1.0E-3*).

#### Poly(A)-tail length estimation

We implemented poly(A)-tail length estimator packages from Nanopolish (110) and Dorado (v0.5.3) to retrieve the length of poly(A)-tails of viral mRNAs. While the 5′- poly(A) leader sequences were counted at the 5′-soft-clipped region of mapped mRNAs allowing one mismatch after three bases of As/Ts.

### Clade determination of the viral isolate

We performed genome alignment of our isolate (MPXV_NRL_4279_2022) with the consensus sequence obtained from dRNA-Seq reads using the MAFFT algorithm (111) with the following settings: gap penalty open: 1.53, offset value: 0.123, and scoring matrix: 200 PAM/*k* = 2. Representative members of each MPXV clade were downloaded from the NCBI viral genome collection based on published data (105, 112). The classification of our sample was determined based on the distance matrix, which was calculated using Geneious software (Supplementary File 1).

## Data Availability

Bam files from CAGE-Seq have been deposited in the European Nucleotide Archive and are available under the Project Accession: PRJEB60061. dRNA-Seq data are available from the PRJEB56841 study.

## SUPPLEMENTAL MATERIAL

File_S1 msphere.00356-24-s0001.csv Distance matrix of aligned MPXV genomes.

Supplemental Figures msphere.00356-24-s0002.pdf Figures S1 to S4.

Supplemental Material msphere.00356-24-s0003.docx Legends to supplemental material and supplemental methods.

Table S1 msphere.00356-24-s0004.xlsx CAGE-Seq peaks detected by TSSr.

Tables S2 and S3 msphere.00356-24-s0005.xlsx TSSs and shape scores.

Table S4 msphere.00356-24-s0006.xlsx TESs.

Table S5 msphere.00356-24-s0007.csv Estimated poly(A)-tail lengths.

Table S6 msphere.00356-24-s0008.xlsx 5'-UTRs.

Table S7 msphere.00356-24-s0009.xlsx 3'-UTRs.

Table S8 msphere.00356-24-s0010.xlsx Novel TSSs found in intergenic regions,

ASM does not own the copyrights to Supplemental Material that may be linked to, or accessed through, an article. The authors have granted ASM a non-exclusive, world-wide license to publish the Supplemental Material files. Please contact the corresponding author directly for reuse.
