# Supplementary material for: Exploring the transcriptomic profile of human monkeypox virus via CAGE and native RNA sequencing approaches
Source: mSphere. 2024 Aug 27;9(9):e00356-24. doi: 10.1128/msphere.00356-24 (PMC11423596; doi:10.1128/msphere.00356-24)
Supplement: Supplemental Figures — Figures S1 to S4. [file msphere.00356-24-s0002.pdf]

Supplementary Figure 1

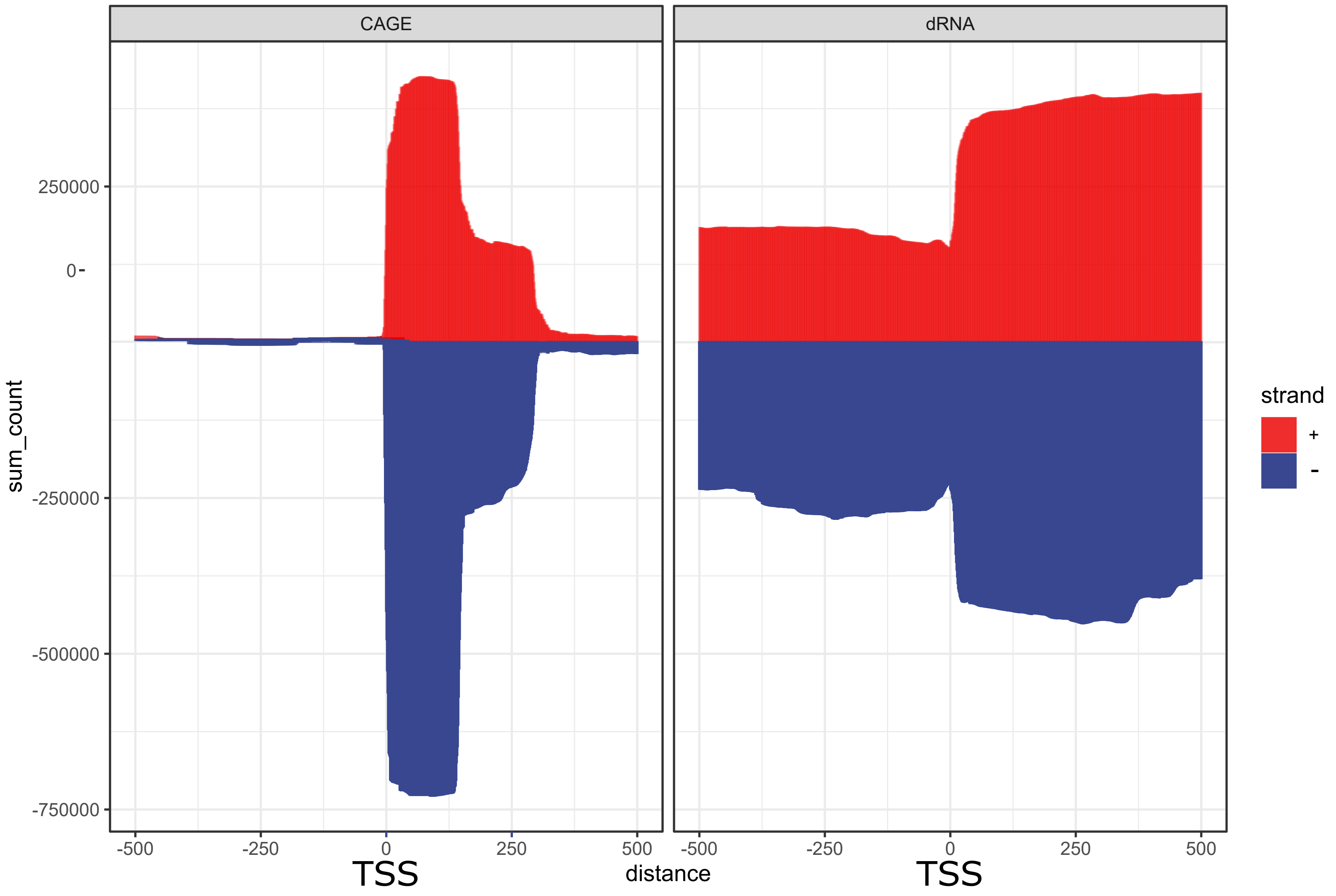

Supplementary Figure 1. Read coverage of CAGE-Seq and dRNA-Seq around the TSSs

This figure illustrates the coverage of CAGE and dRNA -Seq reads in the regions surrounding the TSS positions within a 500-nucleotide window on both sides, separated by strands

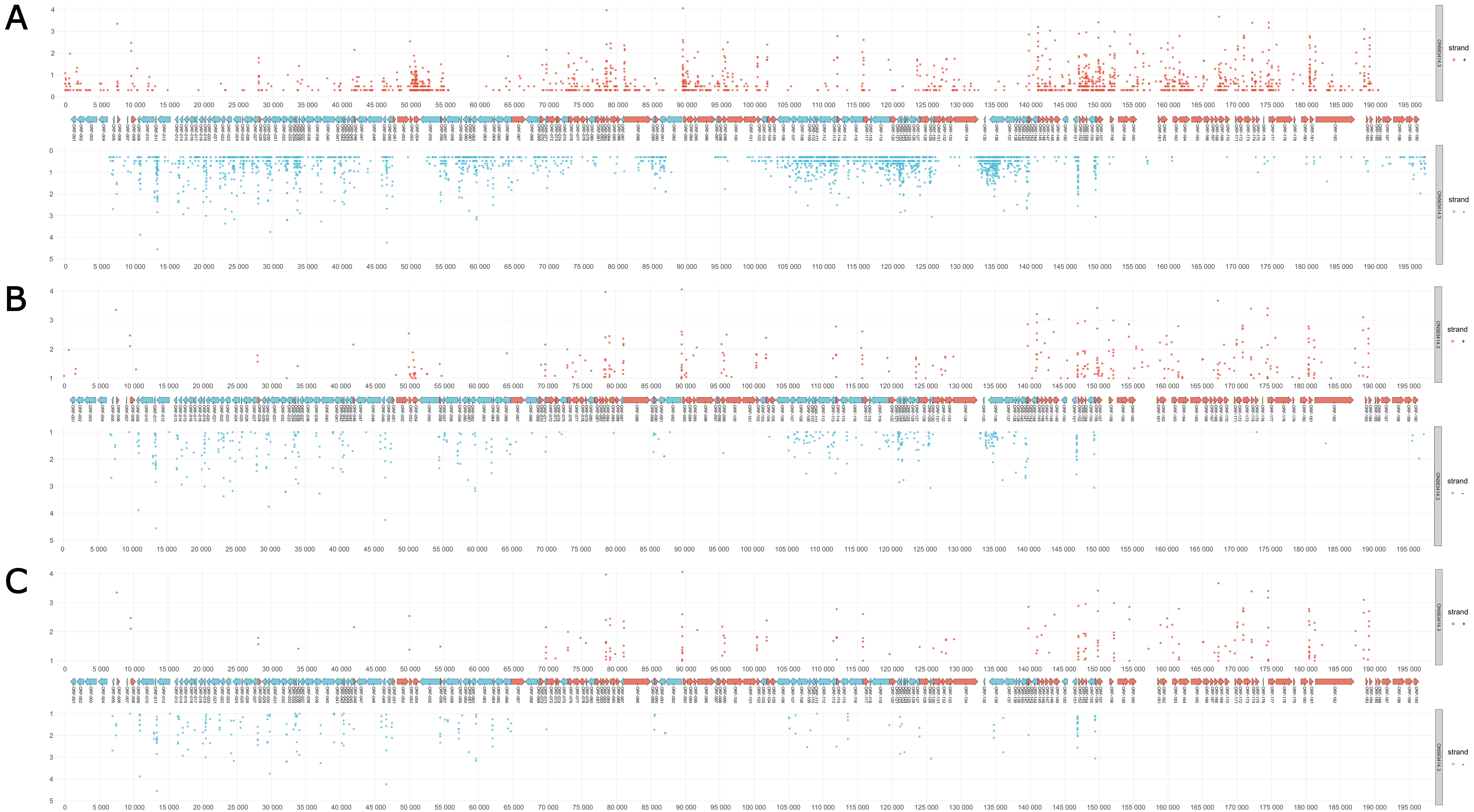

This figure displays the distribution of putative TSS positions following various filtering steps, shown on a logarithmic scale.

A: All putative TSS positions before any filtering (altogether 9,599 TSSs are shown).

B: Putative TSS positions with a CAGE signal of 10 or more (altogether 720 TSSs are shown).

C: Putative TSS positions requiring a minimum CAGE signal of 10, validated by a promoter within a 40 -nucleotide window, and by dRNA-Seq 5'-ends within a 25 -nucleotide window (altogether 401 TSSs are shown).

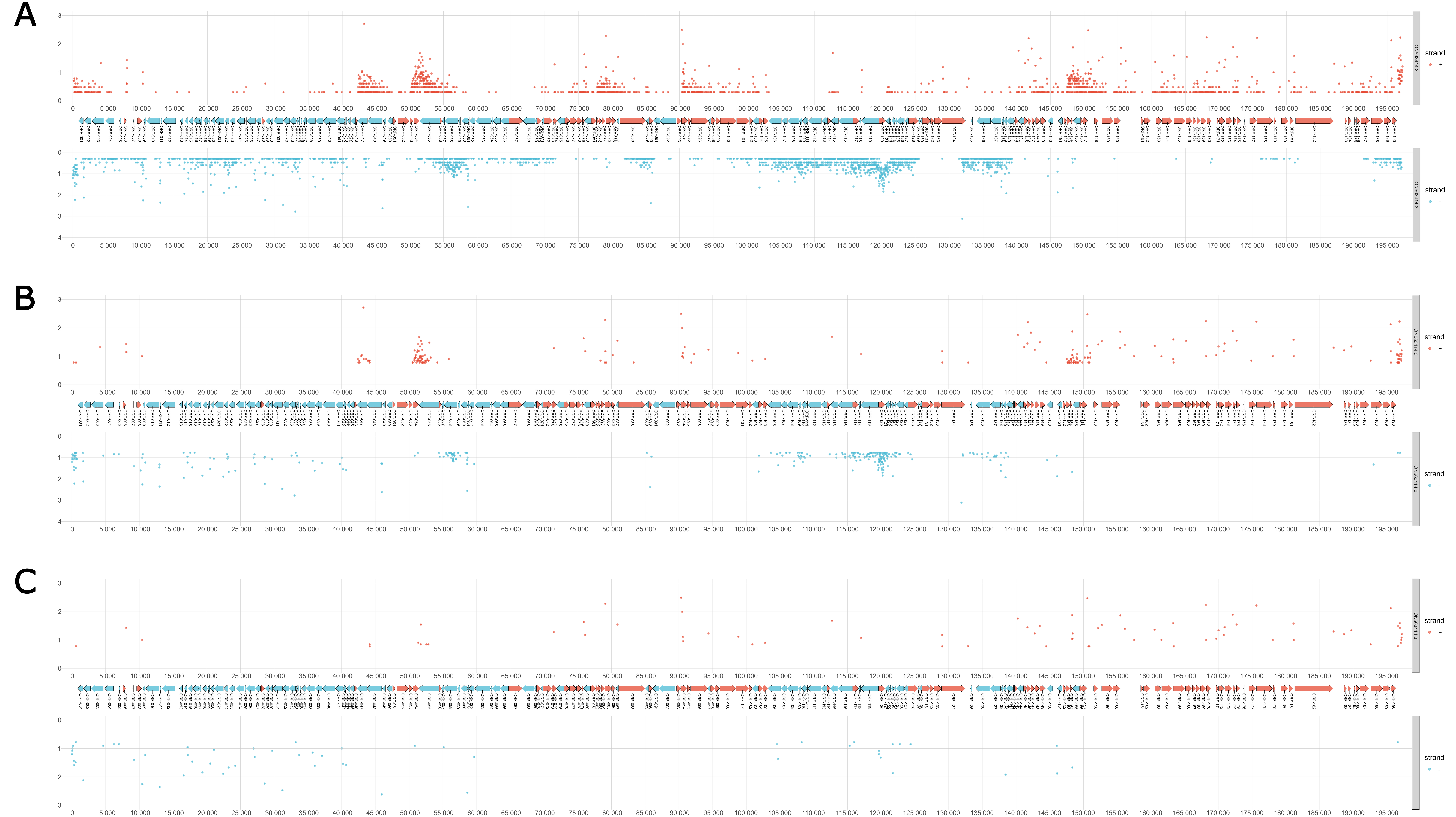

Supplementary Figure 3. Putative TESs detected by LoRTIA

This figure shows the distribution of putative TES positions after various filtering steps, presented on a logarithmic scale.

A: All putative TES positions before any filtering (altogether 3,241 TESs are shown).

B: Putative TES positions confirmed by 6 or more dRNA-Seq reads (altogether 496 TESs are shown).

C: Putative TES positions requiring a minimum dRNA -Seq reads of 6, validated by a poly(A) signal within a 50 -nucleotide window (altogether 135 TESs are shown).

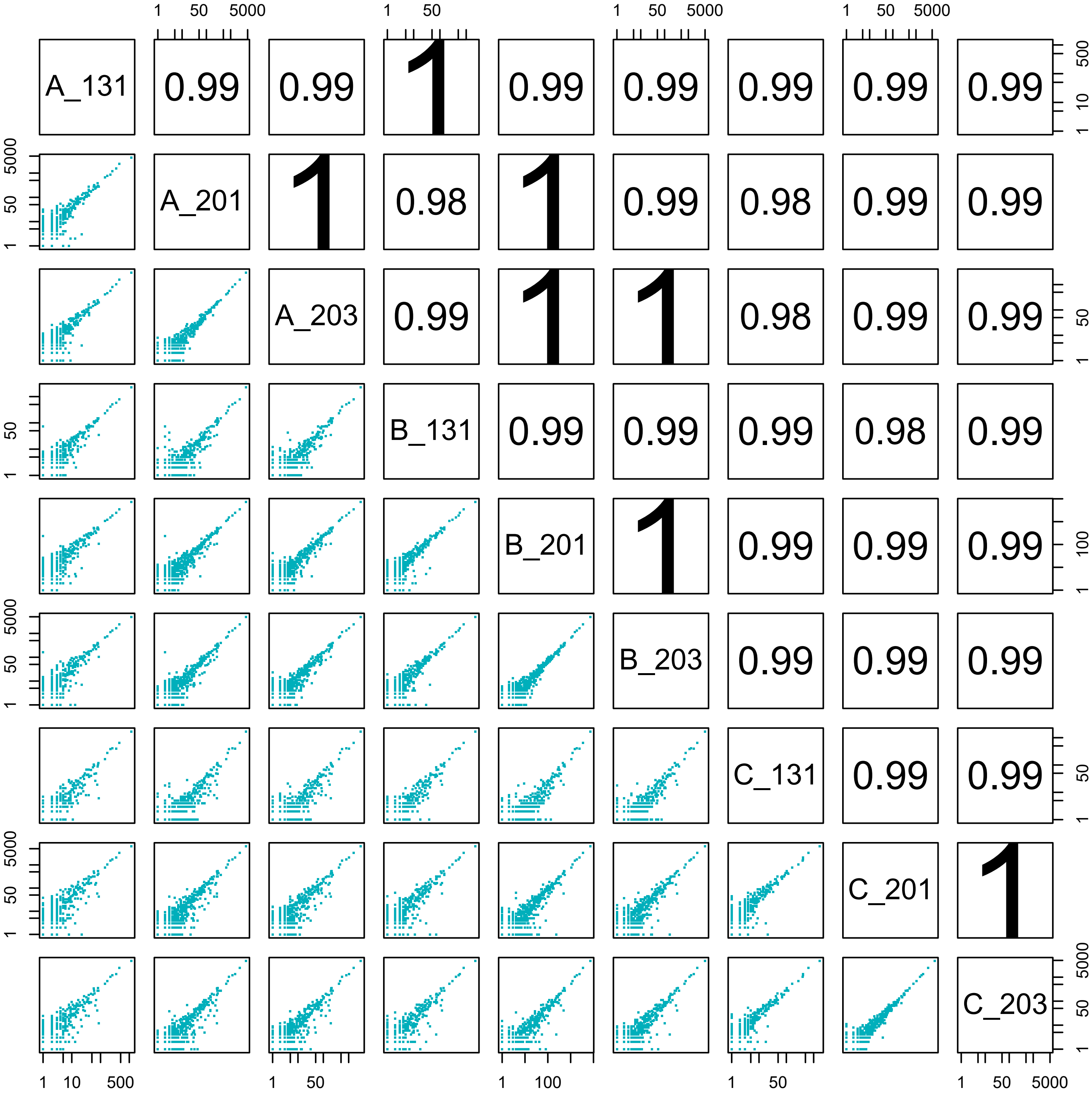

Supplementary Figure 4. Correlation matrix of the three sequenced samples

CAGE-Seq was conducted with three replicates for each of three samples (A, B, C). The plots demonstrate consistency in CAGE-Seq signal positions across all compared bam files. The bam files were all merged into one file.
