# Supplementary material for: Exploring the transcriptomic profile of human monkeypox virus via CAGE and native RNA sequencing approaches
Source: mSphere. 2024 Aug 27;9(9):e00356-24. doi: 10.1128/msphere.00356-24 (PMC11423596; doi:10.1128/msphere.00356-24)
Supplement: Supplemental Material — Legends to supplemental material and supplemental methods. [file msphere.00356-24-s0003.docx]

**LEGEND TO SUPPLEMENTAL MATERIAL**

**Supplementary File 1. Distance matrix of aligned MPXV genomes**

The .csv file contains the distance matrix, names and clade classification of the aligned MPXV sequences.

**Supplementary Figure 1. Read coverage of CAGE-Seq and dRNA-Seq around the TSSs**

This figure illustrates the coverage of CAGE and dRNA-Seq reads in the regions surrounding the TSS positions within a 500-nucleotide window on both sides, separated by strands

**Supplementary Figure 2. Putative TSSs detected by TSSr**

This figure displays the distribution of putative TSS positions following various filtering steps, shown on a logarithmic scale.

**A**: All putative TSS positions before any filtering (altogether 9,599 TSSs are shown).

**B**: Putative TSS positions with a CAGE signal of 10 or more (altogether 720 TSSs are shown).

**C**: Putative TSS positions requiring a minimum CAGE signal of 10, validated by a promoter within a 40-nucleotide window, and by dRNA-Seq 5'-ends within a 25-nucleotide window (altogether 401 TSSs are shown).

**Supplementary Figure 3. Putative TESs detected by LoRTIA**

This figure shows the distribution of putative TES positions after various filtering steps, presented on a logarithmic scale.

**A**: All putative TES positions before any filtering (altogether 3,241 TESs are shown).

**B**: Putative TES positions confirmed by 6 or more dRNA-Seq reads (altogether 496 TESs are shown).

**C**: Putative TES positions requiring a minimum dRNA-Seq reads of 6, validated by a poly(A) signal within a 50-nucleotide window (altogether 135 TESs are shown).

**Supplementary Figure 4. Correlation matrix of the three sequenced samples**

CAGE-Seq was conducted with three replicates for each of three samples (A, B, C). The plots demonstrate consistency in CAGE-Seq signal positions across all compared bam files. The bam files were all merged into one file.

**Supplementary Table 1. CAGE-Seq peaks detected by TSSr**

The table contains the list of the CAGE-Seq signals. (a) This table summarizes all by TSSr detected CAGE-Seq positions along with their p-score and q-score values. (b) dRNA-Seq read’s 5'-positions are listed here with their count values.

(c) The genomic positions of the predicted promoter motifs are listed in a separate column, highlighting the most significant position within a 100 nt binned fraction upstream of a given TSS predicted by FIMO

(d) The table contains the best matching promoter elements upstream of the known ORFs according to their predicted highest q-values. All positions are aligned to the genome: ON563414.3.

**Supplementary Table 2. Clusters of TSSs and list of shape scores**

The table contains clusters of TSSs. The dominant CAGE-Seq signal with count values are listed with the shape-score given by TSSr.Shape-index.

**Supplementary Table 3. List of the most abundant TSSs**

The table shows the top five most abundant CAGE- and dRNA-Seq signals and the adjacent ORF names with their function.

**Supplementary Table 4. List of TESs**

This table presents dRNA-Seq 3'-end positions determined by the LoRTIA toolkit. The U5NU ePAS motif, scanned within a 50 nt binned fraction upstream of a given TES, is listed in a separate column according to their genomic positions.

**Supplementary Table 5. Estimated poly(A)-tail lengths**

The table contains the estimated length of poly(A)-tails of dRNA-Seq reads. (a) The table contains the output data of Nanopolish. (b) The table contains dorado output for poly(A)-tail length estimation.

**Supplementary Table 6. List of 5'-UTRs**

The table categorizes hMPXV ORFs and associated TSSs based on their count values, and their proximity to the ORFs. The 5'-UTRs were determined based on the distance of the canonical TSS belonging to the ORFs**.**

**Supplementary Table 7. List of 3'-UTRs**

The table categorizes ORFs and associated TESs based on their count values, and their distance to the ORFs. The 3'-UTRs were determined based on the distance of the canonical TES belonging to the ORFs**.**

**Supplementary Table 8. Novel TSSs found in intergenic regions**

This table contains a detailed description of the novel genes found in intergenic regions of hMPXV. Novel TSSs and TESs have been identified in the intergenic regions of the hMPXV genome, following the reference sequence ON563414.3. The TSS locations are indicated as LTR (left terminal region) and RTR (right terminal region). The potential lengths of ORFs are calculated based on the coordinates from the first ATG to the subsequent STOP codon, in conjunction with dRNA-Seq reads

**SUPPLEMENTARY METHODS**

**Propagation of cells**

The CV-1 (CCL-70, African green monkey, kidney) cell line, sourced from the American Type Culture Collection (ATCC), was utilized in our study. For experimental purposes, 2 x 10^5^ cells were seeded in 75 cm^2^ tissue culture flasks (CELLSTAR®; Greiner Bio-One GmbH, Frickenhausen, Germany), using Minimum Essential Medium Eagle culture medium (MEM) supplemented with 10% fetal bovine serum (FBS). The cells were grown until they reached approximately 80% confluency (~1.2 x 10^6^ cells) at 37°C in a humid environment containing 5% CO2. Prior to infection, the cell monolayer was rinsed with 1X PBS (Thermo Fisher Scientific, Waltham, MA, USA).

**Propagation of viruses**

The details for collection and isolation of the hMPXV (MPXV_NRL 4279/2022) have been previously described (Kakuk et al., 2023). The virus underwent a single passage on CV-1 cells to yield an adequate quantity of infectious particles. The same batch of working stock was used throughout the experiment. For the infection, 2 ml of hMPXV containing 5 plaque-forming units (pfu)/cell (MOI = 5) was used, diluted with MEM to achieve the necessary concentration. Cells were incubated with the monkeypox inoculum at 37°C for 1 hour, gently shaken every ten minutes. The virus inoculum was then discarded, and the cell monolayer was rinsed with 1X PBS. For each flask, 10 mL of MEM medium was added, supplemented with 2% FBS, 2 mM L-glutamine, and 1% penicillin and streptomycin solution. The cells were then incubated at 37°C for 2, 6, 12, and 24 hours under a 5% CO_2_ humidified atmosphere for RNA sequencing. After incubation, the supernatant was discarded, and the cells were rinsed with PBS. The flasks were then dried and stored at -80°C for subsequent procedures. Cells were then rinsed, scraped into lysis buffer, and transferred into 1.5 mL Eppendorf Tubes® (Thermo Fisher Scientific, Inc.).

**Isolation of total RNA and poly(A) selection**

Cells were collected by centrifugation (1000 x g), then 350µl RA1 lysis buffer (part of the NucleoSpin RNA Kit) and 3.5µl β-Mercapthoethanol (Sigma Aldrich) were added to the samples and then, mixtures were centrifuged at 11,000 x g for 1 min in NucleoSpin Filter tubes (Macherey-Nagel). Filters were discarded, and the lysate was washed using 70% EtOH (350µl) on NucleoSpin RNA Column with centrifugation at 11,000 x g for 30sec. Membrane Desalting Buffer (350µl, from the NucleoSpin RNA Kit) was then added to desalt the membrane, which was finally dried with centrifugation (11,000 x g). Residual DNA was removed using rDNase enzyme. The enzymatic reaction was carried out at room temperature (RT) for 15min. RAW2 Buffer (200µl) was used on the NucleoSpin Filter for inactivation of the enzyme. After a short centrifugation (11,000 x g, 30min) the Filter was placed in a new Eppendorf tube. The next washing step was carried out with RAW3 Buffer (600µl) and centrifugation (11,000 x g, 30min). This step was repeated with 250µl RAW3 Buffer. The purified total RNA samples were eluted from the Filter in 60µl nuclease-free water and they were stored at -80°C.

Polyadenylated RNA was enriched using the Lexogen's Poly(A) RNA Selection Kit V1.5. First, the beads were resuspended and 4µl for each RNA sample was used followed by collection using a magnet. RNAs were resuspended in Bead Wash Buffer (75μl) and then were placed on the magnet, and supernatant was discarded. Beads were resuspended in RNA Hybridization Buffer (20μl). Ten μg from the total RNA samples were diluted to 20µl in nuclease-free water (UltraPure™, Invitrogen) and then they were denatured at 60°C for 1min. RNA samples were mixed with 20µl beads. The mixtures were incubated in a shaker incubator with 1250 rpm agitation at 25°C for 20min. Next, the samples were placed in a magnetic rack. Supernatant was discarded, the tubes were removed from the magnet, the collected samples were resuspended in 100µl Bead Wash Buffer, and finally, they were incubated for 5min at 25°C with 1250 rpm agitation. Beads were resuspended in 12µl nuclease-free water, then kept at 70°C for 1min. After this incubation step, tubes were placed on a magnetic rack and supernatant, containing the polyadenylated fraction of RNA samples were placed to new DNA LoBind (Eppendorf) tubes. This comprehensive approach ensures precise RNA extraction and enrichment crucial for subsequent analyses.

**Cap Analysis of Gene Expression**

To investigate the distribution patterns of transcription start sites (TSSs) in MPXV, we utilized CAGE-Seq with three biological replicates, employing the CAGE™ Preparation Kit (DNAFORM, Japan). We prepared CAGE libraries from 5 µg of total RNA, adhering to the manufacturer's protocol. Briefly, the procedure began by denaturing RNA and RT primer (from the CAGE™ Prep Kit) at 65 °C for 5 minutes. We then synthesized the first cDNA strands using SuperScript III Reverse Transcriptase (Invitrogen), enhancing RT enzyme activity and specificity with a trehalose/sorbitol mixture from the CAGE™ Prep Kit. This was followed by incubation for 30 seconds at 25 °C, and then the RT reaction at 50 °C for 60 minutes. We oxidized diol groups at the 5′-Cap (and 3′-end ribose) using NaIO4, and attached Biotin (long arm) hydrazine. The oxidation involved adding NaOAc (1 M, pH 4.5) and NaIO4 (250 mM) from the CAGE™ Prep Kit to the samples and incubating them on ice for 45 minutes in the dark. Subsequently, 40% glycerol and Tris–HCl (1 M, pH 8.5) from the kit were added. We then mixed NaOAc (1 M, pH 6.0) and Biotin Hydrazine (10 mM) with the samples, biotinylating the oxidized diol residues at 23 °C for 2 hours. Finally, single-stranded RNA was digested by applying RNase I from the CAGE™ Prep Kit, treating it at 37 °C for 30 minutes.

The biotinylated and capped RNA samples underwent Cap-trapping by being combined and bound to pretreated Streptavidin beads, followed by incubation at 37 °C for 30 minutes. They were then placed on a magnetic rack. The beads were sequentially washed with Wash Buffer 1 (twice), then with Wash Buffer 2, and ultimately with Wash Buffer 3, all from the CAGE™ Prep Kit. Following this, cDNAs were detached from the beads by adding Releasing Buffer and incubating at 95 °C for 5 minutes. After briefly placing them on a magnetic rack, the supernatant, which contained the capped cDNAs, was transferred into new tubes. RNase I buffer from the CAGE™ Prep Kit was added to the tRNA-Streptavidin bead, and this mixture was placed on a magnetic rack. The supernatant was then moved to the tubes holding the cDNAs and kept on ice. The samples were treated with an RNase mixture (RNase H and RNase I, both from the CAGE™ Prep Kit) at 37 °C for 15 minutes. Any potential residual RNA was digested with RNase I at 37 °C for 30 minutes.

Streptavidin beads, as part of the CAGE™ Prep Kit, were coated with tRNA and then mixed, followed by a 30-minute incubation on ice and a subsequent 3-minute incubation on a magnetic stand. After removing the supernatant, the beads were washed twice with Wash Buffer 1 from the CAGE™ Prep Kit. The beads were then eluted in the same buffer, to which tRNA was added. The sample volume was reduced using the miVac DUO Centrifugal Concentrator (Genevac). Single-stranded 5′ linkers, containing barcodes, were then attached to the samples at 16 °C for 16 hours using the DNA ligation mixture from the CAGE™ Prep Kit. After purification, the miVac DUO was employed again to further concentrate the samples. Next, the 3′ linker was ligated under similar conditions. Prior to ligation, the 5′ and 3′ linkers were heated to 55 °C and the cDNA samples to 95 °C. The samples were then treated with Shrimp Alkaline Phosphatase (SAP, from the CAGE™ Prep Kit) to remove the phosphate groups from the linkers. This reaction took place at 37 °C for 30 minutes and was terminated at 65 °C for 15 minutes.

The USER enzyme was added to the sample to digest dUTP from the 3′ linker up strand, with the treatment conducted at 37 °C for 30 minutes and halted at 95 °C for 5 minutes. Following this, the barcoded samples were combined and concentrated using the miVac DUO. The synthesis of the second cDNA strands involved the second primer, DNA polymerase, buffer, and 10 mM dNTP, all from the CAGE™ Prep Kit. The denaturation phase was set at 95 °C for 5 minutes, annealing at 55 °C for 5 minutes, and elongation at 72 °C for 30 minutes. The sample mixture was then treated with Exonuclease I enzyme at 37 °C for 30 minutes. A vacuum concentrator was used to completely dry the samples, which were then reconstituted in 10 µl of nuclease-free water. The concentration of single-stranded cDNAs was determined using the Qubit 2.0 and Qubit ssDNA HS Assay Kit. Post-reverse transcription, oxidation, and biotinylation, the samples were purified using RNAClean XP Beads. AmpureXP Beads were employed for further purification after Cap-trapping and releasing, RNase I treatment, 5′ and 3′ linker ligation, SAP and USER treatments, second-strand cDNA synthesis, and Exonuclease I treatment. Libraries with varying barcodes were combined and loaded onto the same flow cells. These libraries were sequenced on an Illumina MiSeq instrument using v3 (150 cycles) and v2 (300 cycles) chemistries. The concentration of the sample was measured using Qubit 4.0 and the 1X dsDNA High Sensitivity (HS) Assay. The quality of the library was verified using TapeStation.
